# Health expenditure efficiency in rural China using the super-SBM model and the Malmquist productivity index

**DOI:** 10.1186/s12939-019-1003-5

**Published:** 2019-07-19

**Authors:** Weilin Liu, Ying Xia, Jianlin Hou

**Affiliations:** 1 Institute of Agricultural Economics and Development, Chinese Academy of Agricultural Sciences, Beijing, China; 2 Institute of Medical Education & National Center of Health Professions Education Development, Peking University, Beijing, China

**Keywords:** Data envelopment analysis (DEA), Health expenditure, Efficiency. Slack-based measure (SBM), Productivity

## Abstract

**Background:**

Health expenditure efficiency (HEE) is an important research area in health economics.

As a large agricultural country, China is faced with the daunting challenge of maintaining equality and efficiency in health resource allocation and health services utilization in the context of rapid economic growth in rural areas. The reasonable allocation of limited rural health resources may be achieved by scientifically measuring the current rural HEE. This subject may help to formulate effective policy or provide incentives for the health sector.

**Methods:**

The combination of a super-slack-based measure (SBM) model with the Malmquist productivity index (MPI) is proposed to evaluate the static health expenditure efficiency (HEE) and dynamic health expenditure efficiency (DHEE) in rural China from 2007 to 2016.

**Results:**

The results show that the HEE and DHEE values exhibit unstable trends over time. The HEE does not follow China’s economic development and presents an average of 0.598 (< 1); and the DHEE presents an average value of approximately 0.949 (< 1), indicating that the DHEE of most provinces is not moving in a desirable direction. The level of technological progress and scale optimization are the main factors hindering total factor productivity (TFP) growth.

**Conclusions:**

The Chinese government could improve the efficiency of rural health resources allocation by improving the rural health service system, optimizing the allocation of material resources and enhancing the level of health of financial resources allocation. The state should continue to moderate policy for different regions. Moreover, scientific and technological advancements should be introduced to improve the scale optimization levels.

## Introduction

Recent years have witnessed an enormous increase in interest in the area of health. Equity and efficiency are important goals pursued by the public health sector [1], and the World Health Organization (WHO) has defined equity, efficiency and utility as the basic criterion for health resource allocation [2]. Efficiency aims to maximize the health level within a certain budget, while equity aims to ensure that the population can enjoy equal medical health service opportunities [3]. Efficiency is a material prerequisite for fairness. In a market economy, health policymakers may pay more attention to efficiency than to fairness, that is to say, it is necessary to optimize health resource allocation and improve health expenditure efficiency (HEE) given a certain budget [4].

HEE is an important research area in health economics. As a large agricultural country, nearly half of China’s population lives in rural areas; therefore, the development of rural health services has an important impact on the overall implementation of the strategy of rural revitalization. Since reform and opening up to the outside world in 1978, the health status and outcomes in China have been significantly improved. According to the World Development Indicators published by the World Bank, life expectancy in China increased from 66.86 years in 1978 to 76.25 years in 2016. Total rural health expenditure increased from 8.81 billion Yuan in 1990 to 344.16 billion Yuan in 2016, approximating a 39-fold increase in health expenditure. However, the situation of rural health care is not optimistic. Rural residents generally have the problems that it is “difficult to see a doctor” and “expensive to see a doctor”, and the phenomena of poverty caused by illness and return to poverty due to illness are becoming increasingly serious. These challenges facing China’s medical system require government authorities to find new solutions when pushing forward reform [5]. The government approved a new scheme for health care reform in 2009 and significantly increased the fiscal budget for medical care to establish a basic medical insurance system for urban and rural residents that efficiently and conveniently provides affordable and safe health services in rural areas. However, the ongoing reform still needs evaluation and correction. How to efficiently deliver enough health services to rural residents and improve the efficiency of the health care system are vital problems for the government authorities.

According to the “central and local fiscal responsibility and expenditure responsibility division reform plan in health China (2018)”, which is explicitly referred to as the “improve primary health services supply efficiency and level” problem, and the “China Rural Revitalization Strategic Plan (2018-2022)”, which was proposed to promote healthy rural construction, service efficiency is one of the important contents of medical health service development at the grassroots level. Especially in rural areas, health resource input is not abundant, and health services tend to have low efficiency and display differences in efficiency across regions. Rural health expenditure efficiency (HEE) is an important topic of study for a variety of stakeholders interested in ameliorating the current situation. On the one hand, policy makers are the voice of citizens and have a fiduciary duty to ensure that national rural health expenditures are sustainable. Policy makers need to know where the current inefficiencies are to formulate effective policy or provide incentives to help the health sector. On the other hand, government authorities need to know whether resources are utilized efficiently, what dimensions can be improved, and what areas are already at their limit. The authorities are also interested in improving the quality of output while utilizing the same amount of resources. The reasonable allocation of limited rural health resources may be achieved by scientifically measuring the current rural HEE. This subject needs the attention of scholars and government departments concerned with the reform and development of rural health.

Recent studies have concentrated on gauging and enhancing health efficiency in practical applications in different dimensions. First, research on the efficiency of national health sectors focuses on calculating efficiency values in different countries, such as OECD countries [6–8], Europe and Central Asia [9]. Second, research has calculated efficiency values in different areas of one country [10–13]. For example, Herwartz & Schley [14] revealed sizeable geographic variation in the allocation of medical services in rural and urban areas in Germany. Carrillo & Jorge [15] compared the efficiency of regional health systems, representing an important incentive for the design and implementation of specific programs aimed at improving the quality of health care services in Spain. Third, scholars study efficiency values according to different hospital characteristics and contexts [16, 17], such as teaching and nonteaching hospitals [18, 19], U.S. federal hospitals [20], religious not-for-profit hospitals [21], and general acute care hospitals in Pennsylvania [22]. In the case of China, Du [13] analyzed the association between quality and efficiency at the national level and in the eastern, central and western regions. There was no significant difference between coastal and noncoastal regions after controlling for other variables [23]. Chu et al. [5] showed that although technical efficiency has improved over time, the hospitals in most Chinese provinces do not perform well in terms of technical efficiency. Another strand of the literature focuses on health spending efficiency and economic growth. Ng [15] revealed that the stage of economic development and the efficiency performance of the hospital do not necessarily go hand in hand.

However, HEE is not a simple summary of different dimensions of health efficiencies, and a more comprehensive study of HEE from a holistic and systematic perspective is needed. Systematic research on HEE in China is still at an elementary stage. The existing research results involve very few fields and are mainly concentrated on the calculation of HEE values. Some scholars [24, 25] analyzed the differences in the efficiency of health expenditure across regions using panel data for 31 provinces in China. Wang & Wang [26] analyzed the efficiency of health expenditure in 13 cities of Beijing, Tianjin and Hebei from 2007 to 2012. However, research on HEE in different provinces of rural China has not been conducted.

In data envelopment analysis (DEA), the accuracy of HEE measurement is closely related to the quality of the input-output indicators selected. First, most studies select health expenditure as the only input indicator [9, 27], and they select the World Development Index as outputs [9, 28], which include years of life expectancy [29], the infant mortality rate [9], and immunizations against measles [6]. Second, some scholars select health expenditure as the only input index and select beds, physicians, nurses, and health institutions [25, 30] as outputs. Third, other studies take health resources, such as hospital beds, health technicians and health institutions, as input indicators and the utilization rate of medical resources, such as the utilization rate of beds, number of outpatient visits and number of inpatients, as outputs [12, 31]. To avoid the correlation of input-output variables, these indicators do not fully include indicators related to health. Thus, we need to aggregate some of the indicators using principal components analysis (PCA) [32].

Regarding the calculation of HEE values, most scholars have adopted traditional or improved DEA models. First, some studies evaluating HEE have employed DEA for OECD countries [6, 33] or developing countries [34, 35]. Other scholars combined the DEA model with other methods. Samut & Cafrı [9] analyzed the health systems efficiency across 29 OECD countries between 2000 and 2010 by two-stage analysis: DEA-Tobit model. Jakovljevic et al. [29] combined DEA with difference-in-differences (DID) to measure health expenditure in Eastern Europe. Stefko et al. [12] measured the regional efficiency of health facilities in Slovakia using DEA and window DEA. Lavado and Cabanda [28] estimated the efficiency of health and education in the Philippines using the DEA, FDH, Malmquist productivity index (MPI) and Tobit methods. Chu et al. [5] investigated the technical efficiency of China’s medical care by proposing a new global generalized directional distance function (GGDDF) approach and Tobit method.

In most previous studies, the traditional DEA model was processed based on radial and angle methods in the efficiency measurement process, and the input-output variable problem cannot be fully addressed by this method. In addition, the current rural health input and output allocation is not at the optimal production scale. Banker-Charnes-Cooper (BCC) [36] DEA assumes variable returns to scale (VRS). Therefore, Liu & Zhang [37] assessed medical service efficiency and identified excessive health problems using the SBM-VRS model. However, as the efficiency values obtained from the SBM-VRS model were between 0 and 1, different decision-making units (DMUs) must be distinguished in the analysis of efficiency. In recent years, super-efficiency DEA models have become an interesting health efficiency research subject. Hsu [9] evaluated the performance of health expenditure in Europe and Central Asia over the period 2005–2007 using the SBM and super-SBM models, respectively. To the author’s knowledge, the super-SBM-VRS model has not been used to evaluate government health expenditure in rural China.

In the DEA approach, the relative HEE is measured among different DMUs at a specific time. However, HEE changes over time cannot be measured with DEA. The MPI measures the dynamic health expenditure efficiency (DHEE). Scholars [8, 9] have used the MPI method to estimate the DHEE in different countries; Lavado & Cabanda [28] measured the dynamic health and education expenditure performance in the Philippines; and researchers [16, 38] have analyzed the dynamic performance of hospitals.

Although a number of studies examine technical efficiency in health [9, 39], only Liu & Zhang [37] pay attention to the productivity and efficiency of health spending in rural China. However, although they can be very insightful, country analyses are rarely used in rural health policy analysis. At the same time, few papers have analyzed the use of PCA, the super-SBM-VRS model, and the MPI as tools for possible application in evaluating the performance of government health spending. This research is among the first attempts to investigate the static and dynamic health expenditure performances across 31 provinces of rural China in such an extensive way.

In summary, the main purpose and innovations of this paper are as follows. First, this research is based on the PCA method to consider more practical input and output indicators, facilitating a better understanding of the relative technical efficiency values in the regional health sector. Second, this paper analyzes the static performance of HEE with the super-SBM-VRS model, which has a high discriminating ability for further ranking efficient DMUs. Finally, to track the HEE performance changes over time, DHEE along with the MPI is computed for consecutive two-year periods during the study period. Meanwhile, we decompose the MPI to locate the sources of productivity growth.

## Research design

### Methodology

#### Improved SBM and super-SBM models

In the Charnes-Cooper-Rhodes (CCR) and BCC models, all outputs are radially expanded to their efficient level by a common expansion factor (or all inputs are radially contracted by a common factor in the input orientation of the model) [40]. Therefore, Tone [41, 42] proposed a slack-based measure (SBM) of efficiency. Unlike the traditional radial DEA model, the slack variables in the SBM model are directly added into the target function to avoid overestimating efficiency. The SBM method is thus non-radial and addresses input/output slacks directly, eliminating the radial and oriented deviation. The SBM model is provided below:

$$ \min \rho =\frac{1-\frac{1}{m}\sum \limits_{i=1}^m{s}_i^{-}/{x}_{ik}}{1+\frac{1}{q}\sum \limits_{r=1}^q{s}_r^{+}/{y}_{rk}} $$

which is subject to.

1 $$ {\displaystyle \begin{array}{l}\sum \limits_{j=1}^n{x}_{ij}{\lambda}_j-{s}_i^{-}={x}_{i0}\\ {}\sum \limits_{j=1}^n{y}_{rj}{\lambda}_j+{s}_r^{+}={y}_{r0}\\ {}\lambda, {s}^{-},{s}^{+}\ge 0\\ {}j=1,2,\dots, \mathrm{n},i=1,2,\dots, m,r=1,2,\dots, \mathrm{q}\end{array}} $$

where *ρ* is the HEE, with 0 < *ρ* ≤ 1; *x* and y are the observed values of DMU inputs and outputs, respectively; *s*^−^ and *s*^+^ represent the input and output slacks for the *DMU* under evaluation; and *λ* is the weight coefficient of the reference DMU.

However, most empirical works on efficiency evaluation research obtain a common result; that is, multiple decision units have the “efficient status” denoted by 100%. Thus, rationally discriminating between these efficient DMUs is important for efficiency ranking and the analysis of influencing factors. To make up for this deficiency of the SBM model, we use the super-SBM model. The super-SBM model addresses excessive input as well as a shortage in output and uses additive models to provide a scalar measure of all inefficiencies [43]. The super-SBM model can be formulated as follows:

$$ \min \delta =\frac{1+\frac{1}{m}\sum \limits_{i=1}^m{s}_i^{-}/{x}_{ik}}{1-\frac{1}{q}\sum \limits_{r=1}^q{s}_r^{+}/{y}_{rk}} $$

which is subject to

2 $$ {\displaystyle \begin{array}{l}\sum \limits_{j=1,j\ne k}^n{x}_{ij}{\lambda}_j-{s}_i^{-}\le {x}_{ik}\\ {}\sum \limits_{j=1,j\ne k}^n{y}_{rj}{\lambda}_j+{s}_r^{-}\ge {y}_{rk}\\ {}\lambda, {s}^{-},{s}^{+}\ge 0\\ {}j=1,2,\dots, n,i=1,2,\dots, m,r=1,2,\dots, \mathrm{q}\end{array}} $$

The SBM model and the super-SBM model assume constant returns to scale (CRS). We can relax and extend the SBM and super-SBM models to the VRS case with the restrictions $\sum \limits_{j=1}^n{\lambda}_j=1$ in Eq. (1) and $\sum \limits_{j=1,j\ne k}^n{\lambda}_j=1$ in Eq. (2), respectively.

#### The Malmquist productivity index (MPI)

An extension of the DEA model is the MPI, which can be applied to measure the total factor productivity (TFP) changes of DMUs between years [44]. The MPI disentangles the total productivity change (tfpch) into technical change (techch) and technical efficiency change (effch) [45]. Technical efficiency change (effch) indicates the degree of progress arising from the innovations that occurred between two periods [46]. Technical change measures the effects of a shift in the production frontier. Technical efficiency change (effch) can be further divided into pure efficiency (pech) and scale efficiency (sech). Pure efficiency evaluates managerial efficiency, while scale efficiency assesses scale suitability for DMUs. If the MPI is greater than 1, the TFP change is positive, and vice versa.

According to Eq. (3) of total factor productivity (TFP), the MPI takes the geometric average from the TFP of the consecutive period.

3 $$ Tfpch= Effch\times Techch=\left( Pech\times \mathrm{Sech}\right)\times Techch $$

We can see the MPI from year “t” to year “t + 1” in Eq. (4):

4 $$ {\displaystyle \begin{array}{l} MPI= TFP=\left({x}^{t+1},{y}^{t+1};{x}^t,{y}^t\right)={\left[\frac{D^{t+1}\left({x}^t,{y}^t\right)}{D^{t+1}\left({x}^{t+1},{y}^{t+1}\right)}\times \frac{D^t\left({x}^t,{y}^t\right)}{D^t\left({x}^{t+1},{y}^{t+1}\right)}\right]}^{\frac{1}{2}}\\ {}\kern2em =\frac{D^t\left({x}^t,{y}^t\right)}{D^{t+1}\left({x}^{t+1},{y}^{t+1}\right)}{\left[\frac{D^{t+1}\left({x}^{t+1},{y}^{t+1}\right)}{D^t\left({x}^{t+1},{y}^{t+1}\right)}\times \frac{D^{t+1}\left({x}^t,{y}^t\right)}{D^t\left({x}^t,{y}^t\right)}\right]}^{\frac{1}{2}}\end{array}} $$

where D is the distant function and its superscripts indicate the time period of efficiency values. The superscripts on x and y indicate the time period for the efficiency value data and represent the outputs and inputs, respectively. *D*^*t*^(*x*^*t*^, *y*^*t*^) and *D*^*t* + 1^(*x*^*t* + 1^, *y*^*t* + 1^) are within-period distance functions.

The MPI has been frequently used to account for changes in policy efficiency and offers many potential advantages for assessing multidimensional environmental impacts that may vary over time. Combining the DEA method with the linear programming method of the parameters, the MPI approach is used to measure the productivity changes from the perspective of multiple inputs and outputs.

### Data sources and indicator selection

The DEA approach does not require functional form assumptions between inputs and outputs and can avoid man-made subjectivity in parameter weighting [47]; one of the most important steps is the right choice of input and output variables. For the improvement in primary health services capacity, the “China Rural Revitalization Strategic Plan (2018-2022)” proposes that the government of each township maintain one township health center and each administrative village, one clinic. Based on the health particularities in rural China, this article considers these two types of health institutions, township health centers and village clinics, as research samples. The indicators used in this paper include two input indicators and eleven output indicators: as inputs, we select healthcare expenditure per capita (PPP) and total expenditure on health (% GDP) (EXP) as rural health spending indicators. Output indicators can be categorized as health institutions, health technical personnel, health facilities, and the utilization rate of health resources. A summary overview and brief definition of these inputs and outputs are given in Table 1.

**Table 1:** Input and output variables

Project	Inputs/Outputs	Abbreviation	Measurement and Explanations	References
Input indicators	Healthcare expenditure per capita (yuan)	PPP	Total rural medical and health expenditure / rural population. This input is related to financial health expenditure per capita in rural areas,	[15, 17, 48]
	Total expenditure on health (% GDP)	EXP	Total rural medical and health expenditure / GDP × 100%. This input represents the degree of government emphasis on health and its fiscal functions.	[8, 15]
health institution outputs	Number of village clinics per thousand rural population (unit)	NC	Number of village clinics / (rural population × 1000). This output reflects the health level of the rural residents near a village clinic.	[30, 37, 49]
	Number of township health centers per thousand rural population (unit)	NTH	Number of township health centers / (rural population × 1000). This output describes the health level of the rural residents near a township health center.	[30, 37, 49]
health technical personnel outputs	Village doctors and assistants per 1000 rural population (ren)	DA	Village doctors and assistants / (rural population × 1000). This output explains the level of human resources in a village clinic.	[9, 22, 23, 37]
	Doctors of township health centers per 1000 rural population (ren)	DTH	Doctors of township health centers / (rural population × 1000). This output indicates the proportion of doctors in the township health centers per 1000 rural population.	[9, 37, 50]
	Licensed (assistant) doctors of township health centers per 1000 rural population (ren/1000)	LDT	Licensed (assistant) doctors of township health centers / (rural population × 1000). This output indicates the technical level of the medical staff in rural areas.	[5, 17, 22, 50]
	Registered nurses of township health centers per 1000 rural population (ren/1000)	NTH	Registered nurses of township health centers / (rural population × 1000). This output indicates the technical level of nurses in rural areas.	[5, 8, 9]
health facility outputs	Beds per 1000 rural population (beds)	BED	Beds of medical institutions / (rural population × 1000). This output indicates the relative number of beds provided by health institutions.	[10, 12, 23, 51, 50]
utilization rate of health resource outputs	Outpatients per 1000 rural population (person-times)	NV	Outpatients in township health centers / rural population × 1000.This output describes the outpatient service level.	[17, 23, 52, 54]
	Number of inpatients (person)	NI	Number of inpatients in township health centers / rural population × 1000. This output describes the inpatient service level.	[9, 23, 49, 50]
	Utilization rate of beds (%)	UB	Actual bed days used / actual available bed days × 100%. The key indicator evaluating bed efficiency.	[12, 17, 22]
	Average duration of hospitalization (day)	ADH	Total number of bed days occupied by discharged persons/total number of discharged persons. This output describes the extent of health care resource utilization.	[12, 13]

To avoid the correlation of variables in the DEA model when there are a significant number of inputs and/or outputs, we used PCA to aggregate some of the indicators. Using PCA reduces the dimensionality of multivariate data and describes the variation in a multivariate data set through linear combinations of the original variables [53]. The results of the Pearson correlation test of the input and output data of rural health services in rural China for 10 years are shown in Table 2 and indicate the possibility of some correlation between input and output variables. We next proceed with the PCA (Tables 3 and 4). The data of the output indicators are z-standardized to obtain a KMO test value of 0.601, and the significance level of Bartlett’s spherical test is sig = 0, indicating that the data are suitable for the main process. Therefore, according to the Kaiser criterion, the eigenvalues are greater than 1, and principal components 1, 2, 3 and 4 account for 84.78% of the total outputs explained. The covariance matrix and component coefficient matrix are shown in Tables 5 and 6.

**Table 2:** Correlation coefficient matrix

Correlation Coefficient	PPP	EXP	NC	NTH	DA	DTH	LDT	NTH	BED	NV	NI	UB	ADH
PPP	1.000	.561^a^	.403^a^	.716^a^	.337^a^	.169^a^	.216^a^	.195^a^	.492^a^	.223^a^	-.215^a^	-.209^a^	.207^a^
EXP	0.561^a^	1.000	.355^a^	.703^a^	.294^a^	-.384^a^	-.143^b^	-.196^a^	-.117^b^	-.204^a^	-.136^b^	-.216^a^	-.204^a^
NC	0.403^a^	.355^a^	1.000	.532^a^	.741^a^	-.185^a^	.215^a^	0.039	.131^b^	-.229^a^	-0.024	-.233^a^	-.200^a^
NTH	0.716^a^	.703^a^	.532^a^	1.000	.320^a^	0.041	-0.062	-.210^a^	0.098	.187^a^	-.274^a^	-.314^a^	.150^a^
DA	.337^a^	.294^a^	.741^a^	.320^a^	1.000	-.197^a^	.166^a^	0.034	.113^b^	-.325^a^	0.078	-.135^b^	-.289^a^
DTH	.169^a^	-.384^a^	-.185^a^	0.041	-.197^a^	1.000	-0.037	0.039	.351^a^	.660^a^	-0.040	0.090	.587^a^
LDT	.216^a^	-.143^b^	.215^a^	-0.062	.166^a^	-0.037	1.000	.887^a^	.598^a^	-.215^a^	0.111	-0.023	-.141^b^
NTH	.195^a^	-.196^a^	0.039	-.210^a^	0.034	0.039	.887^a^	1.000	.636^a^	-.185^a^	.246^a^	0.080	-.130^b^
BED	.492^a^	-.117^b^	0.131^b^	0.098	.113^b^	.351^a^	.598^a^	.636^a^	1.000	.262^a^	.160^a^	.164^a^	.319^a^
NV	.223^a^	-.204^a^	-.229^a^	.187^a^	-.325^a^	.660^a^	-.215^a^	-.185^a^	.262^a^	1.000	-0.103	.294^a^	.958^a^
NI	-.215^a^	-.136^b^	-0.024	-.274^a^	0.078	-0.040	0.111	.246^a^	.160^a^	-0.103	1.000	.774^a^	-.115^b^
UB	-.209^a^	-.216^a^	-.233^a^	-.314^a^	-.135^b^	0.090	-0.023	0.080	.164^a^	.294^a^	.774^a^	1.000	.326^a^
ADH	.207^a^	-.204^a^	-.200^a^	.150^a^	-.289^a^	.587^a^	-.141^b^	-.130^b^	.319^a^	.958^a^	-.115^b^	.326^a^	1.000

^a^significant at the 5% level; ^b^ significant at the 10% level

**Table 3:** KMO and Bartlett test

KMO-Measure of Sampling Adequacy		0.601
Bartlett Test of Sphericity	Approx. Chi-Square	2922.019
	df	55
	sig	0

**Table 4:** Total variance explained

Component	Initial Eigenvalues			Extract Sums of Squared Loadings		
	Total	Variance %	Cumulative %	Total	Variance %	Cumulative %
1	3.066	27.872	27.872	3.066	27.872	27.872
2	2.619	23.811	51.683	2.619	23.811	51.683
3	2.164	19.677	71.360	2.164	19.677	71.360
4	1.476	13.423	84.783	1.476	13.423	84.783
5	0.569	5.172	89.954			
6	0.424	3.854	93.808			
7	0.286	2.599	96.407			
8	0.196	1.786	98.193			
9	0.101	0.919	99.112			
10	0.070	0.637	99.749			
11	0.028	0.251	100

**Table 5:** Covariance matrix

	Component			
	1	2	3	4
NC	-0.558	0.111	0.615	0.406
NTH	-0.112	-0.179	0.790	0.213
DA	-0.590	0.134	0.433	0.475
DTH	0.692	0.178	0.326	-0.088
LDT	-0.290	0.840	0.119	-0.309
NTH	-0.175	0.894	-0.049	-0.323
BED	0.193	0.806	0.356	-0.071
NV	0.900	0.004	0.361	0.093
NI	0.035	0.477	-0.504	0.651
UB	0.439	0.373	-0.460	0.623
ADH	0.871	0.067	0.360	0.090

**Table 6:** Component coefficient matrix

	Component			
	1	2	3	4
NC	-0.319	0.069	0.418	0.334
NTH	-0.064	-0.111	0.537	0.175
DA	-0.337	0.083	0.294	0.391
DTH	0.395	0.110	0.222	-0.072
LDT	-0.166	0.519	0.081	-0.254
NTH	-0.100	0.552	-0.033	-0.266
BED	0.110	0.498	0.242	-0.058
NV	0.514	0.002	0.245	0.077
NI	0.020	0.295	-0.343	0.536
UB	0.251	0.230	-0.313	0.513
ADH	0.497	0.041	0.245	0.074

To smooth the data and meet the input and output data requirements of the DEA model, the inputs are standardized to obtain one DEA model with two input indexes and four output indicators. In addition, since the DEA model requires that the input-output data cannot be negative, the normalized method is used to perform dimensionless processing on the input-output data so that all of data meet the requirements for DEA calculation.

Considering the integrity and availability of data, this paper uses annual panel data on 31 provinces (excluding Hong Kong, Macao, and Taiwan) from 2007 to 2016. The research data for the period 2008–2017 come from the China Statistical Yearbook and the China Health Yearbook.

## Results of empirical analysis and discussion

### Analysis of health expenditure efficiency (HEE) in rural China

As shown in Table 7, we can see that the average HEE value of the 31 provinces is 0.598 and less than 1 for most provinces during the study period, which indicates that the health resource allocation is not optimally configured in rural areas. The provinces with the top five average HEE values are Shanghai, Chongqing, Shandong, Jiangsu and Hunan, which are mainly concentrated in eastern China. Table 7 also shows that the HEE presents an imbalance in the allocation of health resources. In the eastern region, the HEE values range from 0.358 to 1.011, with Hainan having the lowest value of 0.358. In the northeast region, Liaoning and Jilin exhibit the highest and lowest values, respectively. In the central region, Hunan registers the highest HEE value, while Anhui is found to have the lowest value. In the western region, the average HEE values range from 0.302 to 0.946. At the same time, some of the eastern and central regions, represented by Hebei and Jiangxi, and most northeast and west provinces demonstrate inefficient performance for many consecutive years. During the period considered since the health services reform in rural China in 2003, all the above-mentioned regions have faced the tremendous tasks of development and transformation, leading to poor HEE for most provinces. The central government should make reasonable arrangements for the output of health personnel and institutions based on actual local needs to avoid wasting resources and make rational plans for the development of the region.

**Table 7:** Average HEE values of the super-SBM-VRS model for 31 provinces in rural China from 2007 to 2016

Provinces	2007	2008	2009	2010	2011	2012	2013	2014	2015	2016	Average
Beijing (E)	0.513	0.515	0.482	0.534	0.544	0.548	0.534	0.538	0.576	1.015	0.580
Tianjin (E)	0.740	0.728	0.573	0.653	0.613	0.627	0.579	0.563	0.625	0.998	0.670
Hebei (E)	0.858	0.709	0.459	0.525	0.484	0.517	0.501	0.486	0.473	0.543	0.555
Shanxi (C)	1.007	0.627	0.455	1.005	0.473	0.477	0.462	0.419	0.381	0.393	0.570
Inner Mongolia (W)	1.013	0.670	0.475	0.550	0.513	0.533	0.534	0.573	0.629	1.010	0.650
Liaoning (NE)	0.833	0.820	0.589	0.728	0.675	0.720	0.736	0.645	0.645	0.598	0.699
Jinlin (NE)	0.715	0.646	0.442	0.501	0.473	0.490	0.428	0.432	0.372	0.388	0.489
Heilongjiang (NE)	0.812	0.729	0.501	0.574	0.471	0.525	0.541	0.483	0.470	0.514	0.562
Shanghai (E)	1.126	1.013	1.004	1.009	1.031	0.956	1.006	1.009	0.953	1.004	1.011
Jiangsu (E)	0.837	0.788	0.637	1.011	0.759	0.743	0.773	0.836	0.815	1.038	0.824
Zhejiang (E)	0.595	0.548	0.423	0.450	0.331	0.319	0.304	0.299	0.355	1.023	0.465
Anhui (C)	0.674	0.599	0.452	0.485	0.383	0.395	0.391	0.396	0.409	0.476	0.466
Fujian (E)	0.841	0.745	0.578	0.777	0.670	0.734	0.739	0.666	0.643	0.685	0.708
Jiangxi (C)	0.674	0.788	0.462	0.533	0.529	0.701	0.636	0.544	0.547	0.586	0.600
Shandong (E)	1.107	0.933	0.698	1.050	0.872	1.004	1.027	0.891	0.850	1.014	0.945
Henan (C)	0.767	0.763	0.490	0.556	0.476	0.494	0.488	0.487	0.494	0.539	0.555
Hubei (C)	0.846	0.726	0.539	0.695	0.614	0.722	0.789	0.855	0.873	1.018	0.768
Hunan (C)	1.066	1.007	0.581	0.729	0.635	0.730	0.748	0.739	0.853	1.018	0.810
Guangdong (E)	1.070	0.791	0.637	0.927	0.603	0.573	0.556	0.504	0.481	0.489	0.663
Guangxi (W)	0.694	0.611	0.384	0.452	0.397	0.450	0.513	0.489	0.478	0.497	0.497
Hainan (E)	0.605	0.508	0.356	0.389	0.301	0.287	0.293	0.283	0.280	0.280	0.358
Chongqing (W)	1.020	1.016	1.011	1.023	0.707	0.981	1.008	0.820	0.832	1.043	0.946
Sichuan (W)	0.714	0.719	0.515	0.591	0.536	0.669	0.685	0.678	0.685	1.000	0.679
Guizhou (W)	0.413	0.388	0.278	0.333	0.298	0.327	0.377	0.349	0.355	0.393	0.351
Yunan (W)	0.404	0.380	0.277	0.321	0.298	0.333	0.368	0.369	0.364	0.407	0.352
Tibet (W)	0.287	0.305	0.239	0.251	0.503	0.523	0.722	1.009	1.011	1.012	0.586
Shaanxi (W)	0.755	0.607	0.411	0.470	0.440	0.516	0.532	0.534	0.525	0.617	0.541
Gansu (W)	0.352	0.400	0.294	0.357	0.302	0.344	0.344	0.334	0.322	0.352	0.340
Qinghai (W)	0.321	0.317	0.243	0.287	0.311	0.316	0.329	0.334	0.269	0.297	0.302
Ningxia (W)	0.383	0.399	0.275	0.340	0.341	0.339	0.327	0.307	0.314	0.336	0.336
Xinjiang (W)	0.522	0.482	0.333	0.537	0.553	0.589	0.744	0.862	0.861	1.011	0.649
Annual average	0.728	0.654	0.487	0.601	0.520	0.564	0.581	0.572	0.572	0.697	0.598

E, NE, C and W in parentheses refer to the east, northeast, central and west areas, respectively

**Fig. 1:**
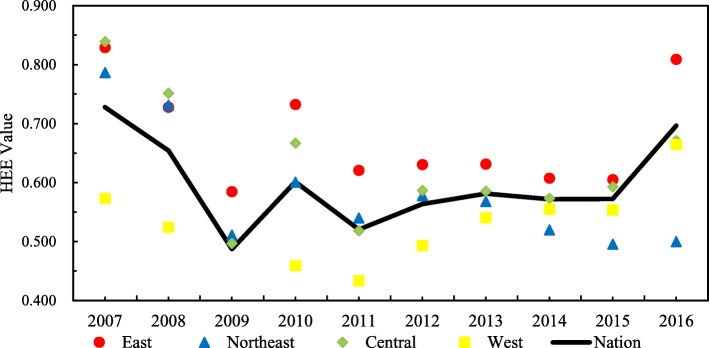
The HEE values of four regions in rural china between 2007 and 2016

Figure 1 shows that from 2007 to 2016, all four regions are inefficient and experience a process of “flexibility” due to the unstable and discontinuous rural health reform policy in different years. Among the four regions, the eastern region obtains the highest efficiency, followed by the central region, with an average HEE value of 0.678. The western region has the lowest value of 0.519. Although China has increased the investment of health funds in rural areas, especially in the western region, there is no corresponding trend of increased efficiency. Figure 1 also shows that the northeast region exhibits a downward trend during 2010–2016, and the gap between the northeast region and the three other regions increases, while the gap narrows between the western region and the three other regions during the study period. The government has recently increased the investment of health funds in the western region, such as by implementing a precision poverty alleviation strategy, for multiple reasons.

The economies of the eastern provinces are relatively developed, and the local governments can allocate more capital to health high-quality resources, relying on the region’s strong economic and technical advantages. The central region has undergone rapid regional development based on its abundant resources. Compared with that of the three other regions, the HEE in rural areas in the northeast region increased only slightly during 2010–2016 due to policy adjustment. In “The Twelfth Five-Year Plan” (2011–2015), the government proposed policies to coordinate urban and rural basic medical insurance and increased the financial support for rural health services; however, health resources are not optimally configured. Government departments should continue to moderate policy, such as the strategies of rural revitalization and precise poverty alleviation, and promote medical and health expenditure efficiency.

### Analysis of dynamic health expenditure efficiency (DHEE) in rural China

As shown in Table 8, the average DHEE value is approximately 0.949 and less than 1 for most provinces, indicating that the DHEE of most provinces is not moving in a desirable direction. The regional differences in dynamic changes are relatively small from 2007 to 2015 except for the period from 2009 to 2010. Then, between 2015 and 2016, the regional differences undergo relatively significant changes, and the HEE scores of most regions (26) increase (> 1). Due to the implementation of basic medical insurance for urban and rural residents, almost all of the provinces experience an improvement in health efficiency. Overall, the health efficiency in 31 provinces of China still requires much improvement. It is difficult to improve efficiency without policy and technical advantages in terms of medical resource allocation capabilities.

**Table 8:** Average DHEE values of 31 provinces in rural China from 2007 to 2016

Provinces	2007–2008	2008–2009	2009–2010	2010–2011	2011–2012	2012–2013	2013–2014	2014–2015	2015–2016	Average
Beijing (E)	1.074	0.995	1.031	1.037	1.002	1.005	1.018	1.022	1.073	1.028
Tianjin (E)	1.012	0.876	1.183	1.015	0.992	0.919	0.981	1.066	1.116	1.014
Hebei (E)	0.791	0.737	1.147	0.869	0.990	0.875	0.921	0.927	1.086	0.919
Shanxi (C)	0.806	0.811	1.305	0.820	0.950	0.943	0.905	0.903	1.075	0.936
Inner Mongolia (W)	0.859	0.950	1.158	0.914	1.035	0.952	1.011	0.990	1.008	0.983
Liaoning (NE)	0.918	0.750	1.283	0.949	1.003	0.950	0.903	0.999	0.922	0.955
Jinlin (NE)	0.860	0.679	1.274	0.927	0.981	0.919	0.977	0.869	1.008	0.932
Heilongjiang (NE)	0.853	0.701	1.166	0.867	1.028	0.942	0.855	0.913	1.055	0.922
Shanghai (E)	0.945	0.911	1.065	1.031	0.967	0.998	0.945	0.941	0.936	0.97
Jiangsu (E)	0.899	0.752	1.332	0.953	1.050	1.041	1.034	0.977	1.084	1.003
Zhejiang (E)	0.905	0.761	1.546	0.983	1.114	1.063	1.043	1.104	1.115	1.053
Anhui (C)	0.814	0.714	0.996	0.722	0.982	0.932	0.950	0.973	1.123	0.903
Fujian (E)	0.966	0.851	1.287	0.869	1.020	0.949	0.930	0.988	1.061	0.984
Jiangxi (C)	1.050	0.737	1.101	0.916	1.002	0.877	0.853	0.946	1.009	0.937
Shandong (E)	0.913	0.807	1.308	0.903	1.032	0.999	0.918	0.973	1.025	0.978
Henan (C)	0.917	0.697	1.144	0.829	0.896	0.886	0.907	0.937	1.017	0.907
Hubei (C)	0.855	0.705	1.177	0.839	1.062	0.938	0.933	0.944	1.038	0.934
Hunan (C)	0.928	0.751	1.197	0.833	0.984	0.933	0.911	0.986	1.049	0.945
Guangdong (E)	0.864	0.779	1.233	0.753	0.910	0.921	0.833	0.948	0.972	0.904
Guangxi (W)	0.888	0.671	1.084	0.745	1.053	1.030	0.880	0.922	0.969	0.906
Hainan (E)	0.800	0.653	1.130	0.776	0.923	0.922	0.900	0.935	0.983	0.882
Chongqing (W)	0.937	0.816	1.205	0.841	1.042	0.981	0.924	0.952	1.097	0.97
Sichuan (W)	0.927	0.760	1.168	0.798	1.017	0.919	0.919	0.938	0.995	0.931
Guizhou (W)	0.884	0.694	1.002	0.790	1.044	0.947	0.827	0.923	1.031	0.897
Yunan (W)	0.860	0.706	1.097	0.856	1.063	1.016	0.948	0.940	1.041	0.94
Tibet (W)	0.992	0.624	1.066	1.164	1.059	1.010	0.983	0.916	1.016	0.969
Shaanxi (W)	0.785	0.673	1.199	0.921	0.997	0.906	0.939	0.939	1.094	0.928
Gansu (W)	0.792	0.680	1.098	0.779	1.086	0.949	0.902	0.910	1.014	0.902
Qinghai (W)	1.060	0.763	1.227	0.968	0.936	0.972	0.994	0.891	1.064	0.979
Ningxia (W)	0.803	0.611	1.080	0.934	0.958	0.905	0.907	0.985	1.028	0.902
Xinjiang (W)	1.006	0.717	1.576	0.977	1.024	1.079	0.977	0.977	1.075	1.026
Annual average	0.898	0.748	1.182	0.884	1.005	0.956	0.932	0.955	1.037	0.949

E, NE, C and W in parentheses refer to the east, northeast, central and west areas, respectively

The average efficiency for each of the 31 provinces in MPI is listed in Table 9. The average tfpch is 0.949, with 5.4% of efficiency resulting from a decline in technological progress and 0.3% from an upward trend in technical efficiency. Five out of 31 (16.12%) provinces have an average tfpch greater than 1, and more than 80% of the provinces show a decreasing TFP. Among them, only 3 provinces show an average techch greater than 1, while 18 provinces exhibit an effch above 1. The decreasing TFP in the majority of the provinces is due to slow technological progress. Eleven out of 31 provinces exhibit a decrease in technical efficiency, of which 5 provinces show an average scale efficiency below 1, while the poor scale efficiency of 6 regions is caused by both inefficient sech and inefficient pech. To improve the TFP in these provinces, resource allocation, technological progress, management level and scale optimization should receive more attention.

**Table 9:** MPI of 31 provinces in rural China from 2007 to 2016

Provinces	effch	techch	pech	sech	tfpch
Beijing (E)	1.007	1.021	1.000	1.007	1.028
Tianjin (E)	1.021	0.993	1.002	1.019	1.014
Hebei (E)	1.006	0.913	1.001	1.006	0.919
Shanxi (C)	0.998	0.938	1.000	0.998	0.936
Inner Mongolia (W)	0.993	0.990	1.000	0.993	0.983
Liaoning (NE)	1.006	0.949	1.003	1.003	0.955
Jinlin (NE)	0.987	0.945	0.989	0.998	0.932
Heilongjiang (NE)	0.992	0.929	1.006	0.986	0.922
Shanghai (E)	1.000	0.970	1.000	1.000	0.970
Jiangsu (E)	1.006	0.997	1.005	1.001	1.003
Zhejiang (E)	1.022	1.030	1.012	1.010	1.053
Anhui (C)	1.003	0.900	1.013	0.990	0.903
Fujian (E)	0.990	0.994	0.993	0.997	0.984
Jiangxi (C)	1.008	0.930	1.007	1.001	0.937
Shandong (E)	1.000	0.978	1.000	1.000	0.978
Henan (C)	0.991	0.915	0.994	0.997	0.907
Hubei (C)	1.000	0.934	1.001	0.998	0.934
Hunan (C)	1.000	0.945	1.000	1.000	0.945
Guangdong (E)	0.964	0.938	0.979	0.984	0.904
Guangxi (W)	0.983	0.922	0.987	0.995	0.906
Hainan (E)	0.958	0.920	0.970	0.988	0.882
Chongqing (W)	1.000	0.970	1.000	1.000	0.970
Sichuan (W)	1.010	0.921	1.018	0.993	0.931
Guizhou (W)	0.999	0.898	1.006	0.993	0.897
Yunan (W)	1.030	0.912	1.032	0.999	0.940
Tibet (W)	1.070	0.906	1.000	1.070	0.969
Shaanxi (W)	1.008	0.921	1.007	1.001	0.928
Gansu (W)	1.012	0.892	1.011	1.001	0.902
Qinghai (W)	1.040	0.941	1.058	0.983	0.979
Ningxia (W)	0.991	0.909	1.007	0.984	0.902
Xinjiang (W)	1.009	1.017	1.024	0.985	1.026
Average	1.003	0.946	1.004	0.999	0.949

E, NE, C and W in parentheses refer to the east, northeast, central and west areas, respectively

The annual averages of DHEE at the provincial level are shown in Table 10. The average tfpch is below 1, and the growth rate of the average DHEE is − 5.1%, suggesting a downward trend with only three years showing an increased TFP during 2007–2016. This means that there is still room for HEE improvement and development. The values for techch and sech were less than 1 for most of the years considered; that is, technological progress and scale efficiency showed a downward trend, leading to low DHEE values. Technological progress is the main driver of the increase in rural HEE and national economic growth and welfare. With the development of China’s economy, the demand for public health services is also increasing. Meanwhile, due to the scattered population and backward infrastructure in rural China, the scale utilization of health resources must receive more attention. The central government should continue to devote more resources and funds to improving medical services in rural areas.

**Table 10:** DHEE averages in 31 provinces of rural China

Year	effch	techch	pech	sech	tfpch
2007–2008	1.009	0.890	1.020	0.990	0.898
2008–2009	0.952	0.785	0.957	0.995	0.748
2009–2010	0.999	1.183	1.007	0.992	1.182
2010–2011	1.016	0.870	1.021	0.996	0.884
2011–2012	1.015	0.990	1.016	0.999	1.005
2012–2013	1.004	0.952	1.000	1.004	0.956
2013–2014	1.017	0.916	1.007	1.010	0.932
2014–2015	0.988	0.966	0.989	0.999	0.955
2015–2016	1.028	1.008	1.021	1.007	1.037
Average	1.003	0.946	1.004	0.999	0.949

**Fig. 2:**
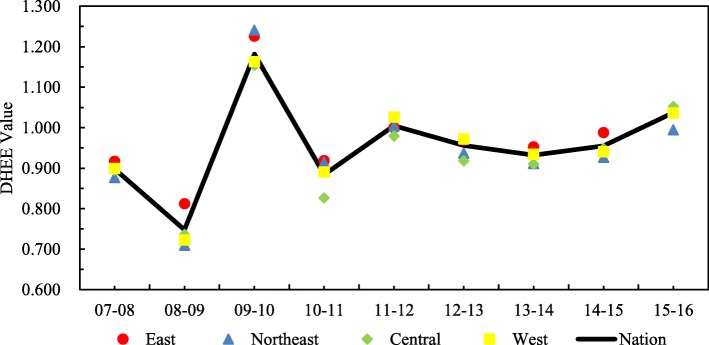
The average DHEE values of four regions in rural China over time

Figure 2 presents the average DHEE values of different regions in rural China over time. Of the DHEE values in the four regions, those of the eastern region are the highest from 2007 to 2009 and 2010 to 2015. However, the DHEE values of the northeast region are the highest in 2009–2010, and the DHEE values of the central region are the highest in 2015–2016. Figure 2 also shows that the DHEE value peaks in 2010 because health system reform promotes technological advancement [30, 51]. The results also indicate that the average DHEE values of the western region gradually catch up with those of the other regions during the study period. This narrowing of the gap between regions shows that the central government has increased its investment in funds and resources, and local governments in the western region have attributed greater importance to medical management and technological progress [54]. The DHEE values of all four regions increase gradually during 2013–2016 because during this period, the basic medical insurance system for urban and rural residents and supply-side reforms are beginning to bear fruit.

## Conclusions

This study shows that the HEE and DHEE values exhibit unstable trends over time; meanwhile, the efficiency in the rural areas of most provinces is low and has great potential for improvement. The results show that the average level of overall HEE is 0.598 and the average DHEE value is 0.949 during the sample period, which is due to the unstable and discontinuous rural health reform policy. Although China has increased the investment of health funds in rural areas, especially in the western region, there is no corresponding trend of increased efficiency. In the blind pursuit of rapid economic development, improvement at the HEE and DHEE levels has been neglected. The health level needs further development, and resource allocation requires further optimization to avoid the waste of resources.

A serious imbalance is observed between provincial and regional HEE. This study shows that the HEE varies considerably across provinces: Shanghai, Chongqing, Shandong, and Jiangsu provinces, mainly concentrated in eastern China, experienced consistently high efficiency scores, whereas Qinghai, Xinjiang, Gansu, and Guizhou in western China experienced lower HEE values. The comparable estimates of health efficiency in the four regions likely reflect the limited health options available in rural areas due to geographic barriers and different policy support.^1^ The economies of the eastern provinces are relatively developed, and the local governments can allocate more capital to high-quality health resources because of the region’s strong economic and technical advantages. The government has recently increased its investment of health funds in the western region, such as by implementing a precision poverty alleviation strategy. The central region has undergone rapid regional development based on its abundant resources. Compared with the other three regions, the eastern region has relatively reasonable resource allocation due to policy support and geographic advantage. The development of macrolevel planning has a positive effect on the optimization of health efficiency in rural China. Government departments should continue to moderate policies for different regions, such as rural revitalization and precise poverty alleviation strategies, and promote HEE.

Based on the measured DHEE levels, the average effch, techch, pech, sech and tfpch values are 1.003, 0.946, 1.004, 0.999 and 0.949, respectively. The DHEE levels undergo two stages: a stage before the year 2009 and a stage after. DHEE values peaked during 2009–2010 due to a new scheme for health care reform in 2009, and the techch and pech values during this period exceeded 1. The average tfpch reflects a process of “flexibility” during 2011–2015, but was higher than 1 in the subsequent years. We find an increase in technical efficiency and pure efficiency and a decline in TFP, technological progress and scale efficiency. Therefore, technological progress and scale optimization levels need to be improved. Among them, technological progress plays a very important role in the country’s economic growth and welfare, as it is the main driving force of TFP. On the one hand, there are many differences in technological progress across regions, so the government needs to formulate economic development policies according to the local conditions and introduce advanced science and technology. On the other hand, the government needs to grasp the positive aspects common to the areas within the province and actively promote advancement to achieve scale effects.

## Data Availability

The data supporting the findings of this study are available from the Census and Statistics Department of the Ministry of Public Health in China.

## Abbreviations

ADH: Average duration of hospitalization
BCC: Banker-Charnes-Cooper
BED: Beds per 1000 rural population
CCR: Charnes-Cooper-Rhodes
DA: Village doctors and assistants per 1000 rural population
DEA: Data envelopment analysis
DHEE: Dynamic health expenditure efficiency
DTH: Doctors of township health centers per 1000 rural population
effch: Technical efficiency change
EXP: Total expenditure on health
HEE: Health expenditure efficiency
LDT: Licensed (assistant) doctors of township health centers per 1000 rural population
MPI: Malmquist productivity index
NC: Number of village clinics per thousand rural population
NI: Number of inpatients
NTH: Number of township health centers per thousand rural population
NTH: Registered nurses of township health centers per 1000 rural population
NV: Outpatients per 1000 rural population
PCA: Principal components analysis
pech: Pure efficiency
PPP: Healthcare expenditure per capita
SBM: Slack-based measure
sech: Scale efficiency
techch: Technical change
techch: Technical efficiency change
TFP: Total factor productivity
tfpch: Total productivity change
UB: Tilization rate of beds
VRS: Variable returns to scale
WHO: The World Health Organization

## Footnotes

1 lndeed, there are many potential confounders that could cause one region to be more efficient than another, including social factors, population health factors, environmental factors, and other economic factors. These factors may impact HEE, and this issue warrants further investigation
